# Brain tissue pulsation responses to a low‐level expiratory resistance manoeuvre: A pilot healthy volunteer study

**DOI:** 10.1113/EP093543

**Published:** 2026-10-25

**Authors:** Jennifer K. Nicholls, Emma M. L. Chung, Jonathan Ince, Edward Pallett, Jatinder S. Minhas, Andrea Lecchini‐Visintini

**Affiliations:** ^1^ Cerebral Haemodynamics in Ageing and Stroke Medicine (CHiASM) Research Group, Division of Cardiovascular Sciences University of Leicester Leicester UK; ^2^ University Hospitals of Leicester NHS Trust Leicester UK; ^3^ Division of Cardiovascular Sciences, British Heart Foundation Centre Research Excellence University of Leicester Leicester UK; ^4^ School of Life Course and Population Sciences Faculty of Life Sciences and Medicine, King's College London London UK; ^5^ Clinical Engineering University Hospitals of Leicester NHS Trust Leicester UK; ^6^ NIHR Leicester Biomedical Research Centre, Leicester British Heart Foundation Centre of Research Excellence Glenfield Hospital Leicester UK; ^7^ School of Electronics and Computer Science University of Southampton Southampton UK

**Keywords:** brain tissue pulsations, expiratory resistance, transcranial tissue Doppler ultrasound

## Abstract

Pulsatility of the brain has been hypothesised to be linked to intracranial pressure (ICP), although the nature of this relationship remains poorly understood. We used a low‐level expiratory resistance manoeuvre to characterise brain tissue pulsation (BTP) responses in a pilot healthy volunteer study. BTPs were measured using a transcranial tissue Doppler ultrasound prototype in 10 healthy volunteers at rest and during the manoeuvre. Measurements of mean arterial pressure (MAP), pulse pressure (PP), and heart rate (HR) were also recorded. One‐way repeated‐measures ANOVA compared changes between baseline, each phase of the manoeuvre, and recovery. Bulk BTP amplitude increased significantly during the initial phase compared with baseline (*P* = 0.004). A non‐significant decrease was observed as the manoeuvre continued, with values decreasing below baseline during the late phase, followed by an increase above baseline during recovery. HR increased significantly during the late phase (*P* = 0.015). No significant changes were observed in MAP or PP. Bulk BTP amplitude demonstrated a dynamic response during low‐level expiratory resistance, characterised by an initial increase followed by a non‐significant decrease over the remainder of the manoeuvre. The physiological mechanisms underlying these observations remain uncertain. Future studies incorporating direct ICP measurements and complementary physiological monitoring are needed to clarify the relationship between BTPs and intracranial dynamics.

## Abstract (highlights)

- **What is the central question of this study?**
  How do brain tissue pulsations (BTPs) respond to a low‐level expiratory resistance manoeuvre in healthy adults?
- **What is the main finding and its importance?**
  During the initial phase of the manoeuvre, bulk BTP amplitude significantly increased. This was followed by a non‐significant decrease in bulk BTP amplitude below baseline as the manoeuvre continued into the late phase. These findings suggest that changes in bulk BTP amplitude can be measured using transcranial tissue Doppler ultrasound during low‐level expiratory resistance.

## 1 INTRODUCTION

The Monro–Kellie doctrine, based on the original observations of Monro (1783) and later expanded by Kellie (1824), proposes that, for normal brain homeostasis, the volumes of the brain parenchyma, cerebrospinal fluid, and intracranial blood must remain approximately constant within the fixed volume of the skull (Monro, 1783) (Kellie, 1824). A change in one component must be offset by compensatory adjustments in other components (Kim et al., 2012) to prevent rises in intracranial pressure (ICP), which can compromise cerebral perfusion pressure (Bothwell et al., 2019; Craven & Reddy, 2016). Whilst there have been recent proposed revisions to this doctrine (Benson et al., 2023; Brasil et al., 2025; Mangalore et al., 2019; Wilson, 2016), the interplay of these three components remains generally accepted.

Deranged ICP has been observed in many pathological states, such as idiopathic intracranial hypertension (Wakerley et al., 2020), intracranial haemorrhage (McGurgan et al., 2020), and traumatic brain injury (Patel et al., 2023). In traumatic brain injury, sustained elevated ICP is associated with worse clinical outcome and increased mortality risk (Badri et al., 2012; Marmarou et al., 1991; Schreiber, Aoki, Scott & Beck, 2002), highlighting the importance of ICP monitoring. There are multiple ICP monitoring methods available, but the type chosen depends on a patient's clinical condition and the associated risks and benefits of each device (Mathur et al., 2025). Commonly used invasive methods include external ventricular drains and subarachnoid bolts, which are connected to pressure transducers that enable direct measurement of ICP. More recently, there has been growing interest in non‐invasive ICP monitoring techniques, such as transcranial Doppler (Rajajee, 2024) and magnetic resonance imaging, including the measurement of brain motion using amplified magnetic resonance imaging (Holdsworth et al., 2016; Kumar et al., 2025).

The brain parenchyma pulses with each cardiac cycle (Alharbi et al., 2020; Ince et al., 2020; Nicholls et al., 2023; Wagshul et al., 2011), and given its importance within the Monro–Kellie doctrine, it has been hypothesised that brain motion may be correlated with ICP (Holdsworth et al., 2016; Nicholls et al., 2025; Saindane et al., 2018). Increases in intrathoracic pressure are known to influence ICP, as demonstrated in studies of positive end‐expiratory pressure (PEEP) and the Valsalva manoeuvre, via elevation of central venous pressure and cerebral blood volume, and reductions in central venous outflow (Chen et al., 2016; Luecke & Pelosi, 2005; Tiecks et al., 1995; Zunino et al., 2024). Several magnetic resonance imaging studies have assessed changes in brain displacement over the cardiac cycle during interventions associated with alterations in ICP. These studies reported reduced brain displacement during the Valsalva manoeuvre and increased displacement following cerebrospinal fluid removal (Holdsworth et al., 2016; Kumar et al., 2025; Saindane et al., 2018). Taken together, these observations suggest that brain tissue motion may be influenced by the interplay between ICP, cerebral blood volume, and venous outflow, although the nature of these relationships remains poorly understood.

In this study, we used a low‐level expiratory resistance manoeuvre, performed using a whistle containing a PEEP valve, to explore brain tissue pulsation (BTP) responses measured using transcranial tissue Doppler (TCTD) in a pilot study of healthy volunteers. This approach provides an opportunity to investigate dynamic BTP responses using a non‐invasive, real‐time ultrasound‐based technique. Monitoring of blood pressure (mean arterial pressure (MAP) and pulse pressure (PP)) and heart rate (HR) was performed to assess systemic effects. We hypothesised that low‐level expiratory resistance would result in measurable changes in bulk BTP amplitude.

## 2 METHODS

### 2.1 Ethical approval

Healthy adult participants were recruited to the study from staff and students at the University of Leicester, following a protocol approved by the University of Leicester's Medicine and Biological Sciences Research Ethics Committee (approval number 42407), in accordance with the *Declaration of Helsinki* (2013). The study conformed to the standards set by the *Declaration of Helsinki*, except for registration in a database. Written informed consent was received from all participants. Subjects self‐reporting any previous history of brain injury or head trauma, previous intracranial surgery, spinal injuries, herniated discs, concussion, ocular trauma, visual disturbances, glaucoma, orthostatic hypotension, arterial hypotension or any previous cardiovascular health issues were excluded. Participants were requested to refrain from caffeine, vigorous exercise, smoking, alcohol consumption and heavy meals for at least 2 h prior to their appointment.

Measurements were recorded in a quiet, temperature‐controlled laboratory. Participants were asked to lie supine on a hospital bed with an approximate head height of 20 degrees with their eyes closed to prevent motion artefacts.

TCTD measurements were recorded from approximately 1 cm above the centre of the left eyebrow of each participant, using a 2 MHz transcranial Doppler probe from Spencer Technologies (Medway, MA, USA), connected to our acquisition prototype Brain Tissue Velocimetry (Brain TV; Nihon Kohden, Tokyo, Japan). The TCTD probe was held in a fixed orientation for the duration of the study, and cables were secured to reduce motion artefacts. Continuous blood pressure readings were obtained using a Finometer system (Finapres Medical Systems, Enschede, Netherlands), with an appropriately sized finger‐cuff placed around the participant's right middle finger. The Finometer was calibrated prior to each recording using a standard inflatable arm‐cuff device (OMRON Model 705IT; OMRON Corp., Kyoto, Japan). A three‐lead electrocardiogram (Life Scope monitor, Nihon Kohden) estimated HR. End‐tidal carbon dioxide was not measured to prevent the nasal cannula interfering with the equipment set up. All physiological monitoring data were recorded using a sampling rate of 500 Hz. A schematic illustration of the set‐up is given in Figure 1.

**FIGURE 1:**
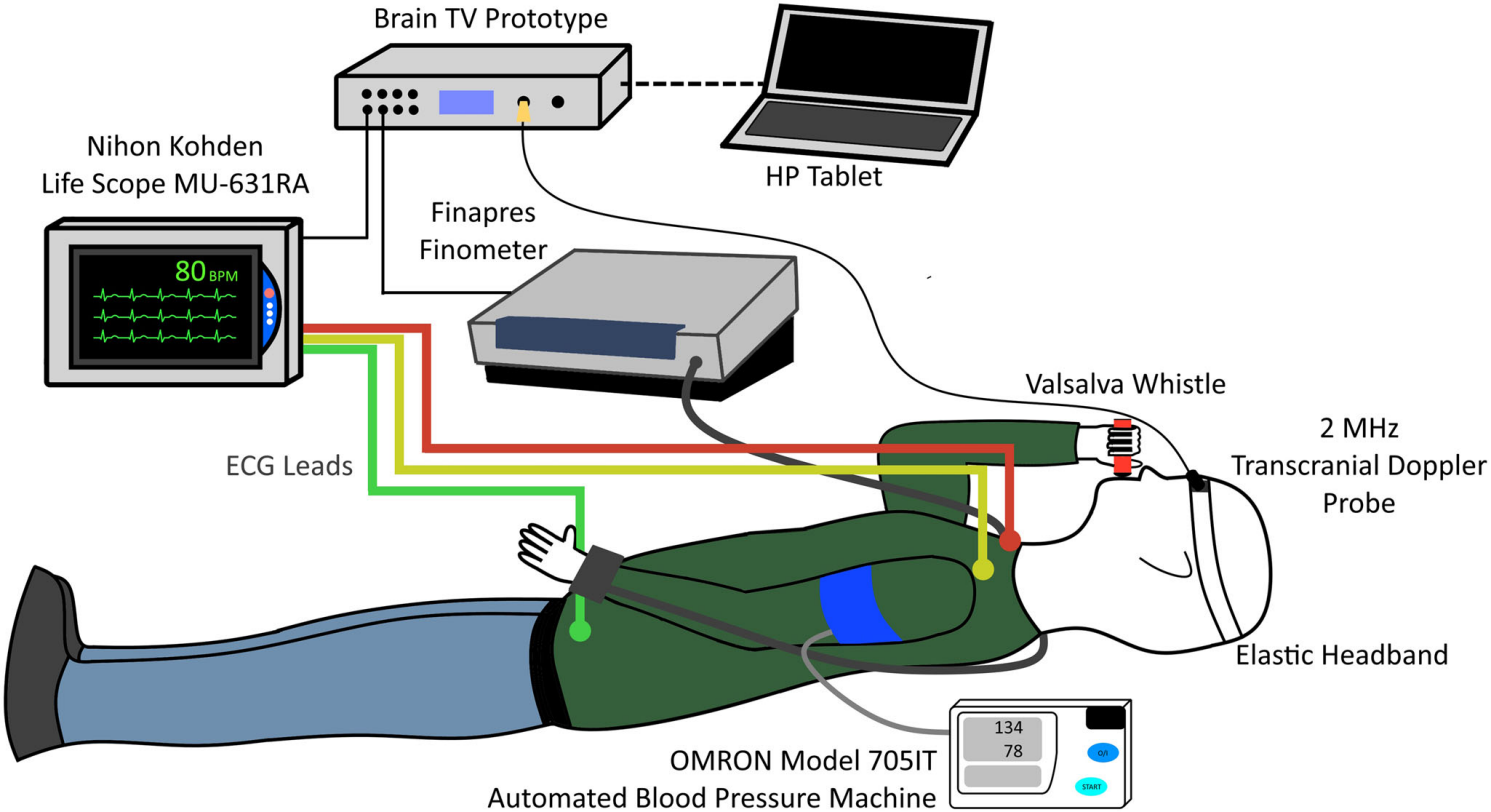
Schematic diagram showing the expiratory resistance manoeuvre set‐up. Reproduced from Nicholls (2024) PhD Thesis, University of Leicester, with permission.

For the expiratory resistance manoeuvre, participants held a whistle in their right hand, which was developed ‘in‐house’ by researchers at the University of Leicester and contained a PEEP valve (Laerdal Medical, Stavanger, Norway) that released at a fixed pressure (∼14.7 mmHg). Participants practiced the expiratory resistance once before data acquisition. Each participant then completed one expiratory resistance as part of the study protocol, including baseline and recovery periods. Participants were asked to breathe normally for the first 58 s of the recording. At 58 s, participants took a normal inspiratory breath. At 60 s, participants exhaled for one prolonged breath for 15 s into the whistle, and this was counted as the expiratory resistance manoeuvre. The recording continued for a further minute, and this was counted as the recovery phase. Following the recovery period, a blood pressure measurement was taken from the participant's left arm.

### 2.2 Data processing

All recordings were processed and displayed using a graphical user interface (GUI) developed ‘in‐house’ in MATLAB (R2022a) (MathsWorks Inc., Natick, MA, USA).

Data processing of BTP recordings has been previously detailed (Nicholls et al., 2023). Down‐sampling of the TCTD data and estimation of the tissue velocity at each depth was performed. BTP signals were filtered using a 0.5–50 Hz bandpass filter to remove respiration, head motion, and high frequency noise. For each recording, a bulk BTP signal was calculated by averaging BTP across all depths with a good BTP signal, which represents collective displacement over the cardiac cycle. The absolute difference between the peak and trough of this bulk BTP signal for each cardiac cycle was used to estimate beat‐to‐beat bulk BTP amplitude using MATLAB (R2022a).

Instantaneous HR was estimated by calculating the mean cardiac frequency based on each subject's electrocardiogram R‐wave interval. A BTP signal from the GUI was provided for every heartbeat, with each value assigned to the midpoint of the R‐R interval. Values at R‐R intervals identified as artefacts were discarded prior to analysis. The remaining sequences were resampled over a uniform time grid (d*T* = 0.5 s) using interpolation with a cubic method.

All BTP recordings were visually inspected to identify any intervals with artefacts. Artefacts were defined as any noticeable transient perturbations not regularly repeating with the cardiac cycle.

### 2.3 Statistics

For each healthy volunteer, a 21 s interval of baseline data, 3 × 5 s fixed intervals during the expiratory resistance manoeuvre (noted as the initial, early and late phases of the manoeuvre), and a 20 s recovery interval were considered for analysis. A time series for a representative recording is shown in Figure 2, including indication of the intervals taken forward for analysis.

**FIGURE 2:**
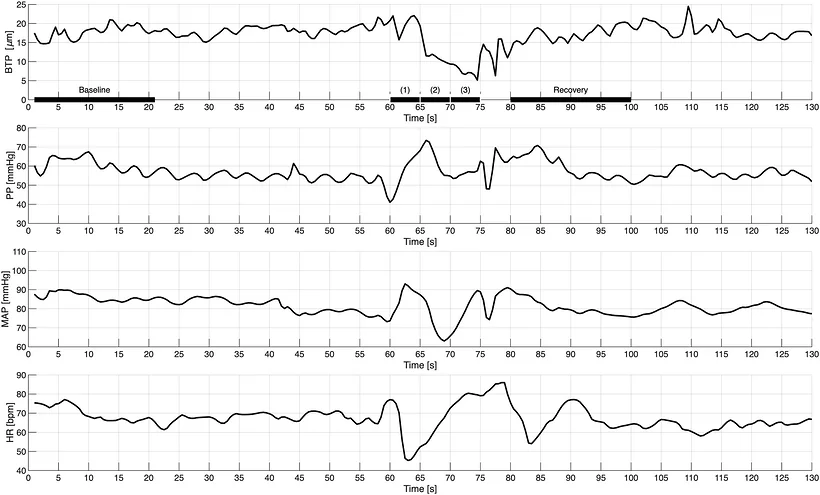
Time series of bulk brain tissue pulsation (BTP) amplitude, pulse pressure (PP), mean arterial pressure (MAP), and heart rate (HR) recorded from a representative 24‐year‐old male who performed the expiratory resistance manoeuvre. Horizontal bars in the BTP panel indicate the five analysis windows: baseline (1–21 s), 1 (60–65 s), 2 (65–70 s), and 3 (70–75 s) and recovery (80–100 s). 1 = initial; 2 = early; 3 = late phase of the manoeuvre.

Baseline was represented by the mean of the beat‐to‐beat average of variables over the baseline interval. The expiratory resistance manoeuvre was represented by the mean of the beat‐to‐beat average of variables over the three phases of the manoeuvre. Recovery was represented by the mean of the beat‐to‐beat variables over the recovery interval. The variables chosen for analysis were PP, MAP, HR, and bulk BTP amplitude.

Data were imported to GraphPad Prism (GraphPad Software, Boston, MA, USA) for statistical analysis. Bulk BTP amplitude was non‐normally distributed in our cohort; all other variables were normally distributed. Comparisons between baseline and the expiratory resistance manoeuvre periods for bulk BTP amplitude were made using a non‐parametric one‐way repeated‐measures ANOVA (Friedman's test), followed by Dunn's *post hoc* correction for multiple comparisons. For HR, PP, and MAP, changes over time were analysed using a parametric one‐way repeated measures ANOVA. If the assumption of sphericity was violated, the Greenhouse–Geisser correction was applied. *Post hoc* comparisons with baseline were performed using Dunnett's multiple‐comparison correction.

Sex‐based differences were assessed using an independent sample Student's *t*‐test (Welch's correction), with Bonferroni correction applied for multiple comparisons.

Median and interquartile range (IQR) are reported for non‐normally distributed variables, while mean and standard deviation (SD) are reported for normally distributed variables. A significance threshold of *P *< 0.05 was applied.

Time series Δ changes in the median and IQR for the cohort are given in Figure 3.

**FIGURE 3:**
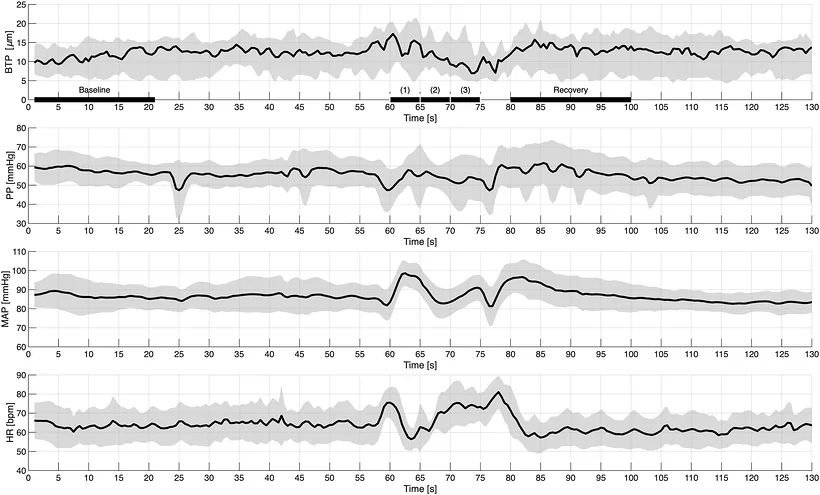
Group time series of brain tissue pulsation (BTP) amplitude, pulse pressure (PP), mean arterial pressure (MAP), and heart rate (HR) across 10 participants during the expiratory resistance manoeuvre. For BTP, the black line denotes the group median, and the shaded region denotes its 95% bootstrap confidence interval (10,000 resamples). For PP, MAP and HR, the black line denotes the group mean and the shaded region denotes its 95% confidence interval. Horizontal bars in the BTP panel indicate the five analysis windows as defined in Figure 2.

## 3 RESULTS

Ten healthy volunteers (50% female), aged 22–52 years (median age: 24.5 years), participated in the study. Good quality BTP signals were recorded from the forehead for the first 27 depths (74 mm) for all subjects. Beyond this depth, visualisation of BTPs was impaired due to limited penetration of the ultrasound beam.

For bulk BTP amplitude, the overall effect of time was significant (Friedman χ^2^(4) = 14.17, *P* = 0.007) (see Table 1 and Figure 4). *Post hoc* testing showed that median bulk BTP amplitude increased significantly from baseline by 2.7 µm during the initial phase of the manoeuvre (*P* = 0.004). Following the initial increase, bulk BTP amplitude progressively declined across the manoeuvre and fell below baseline values during the late phase; however, these changes were not statistically significant. In recovery, bulk BTP amplitude increased above baseline.

**TABLE 1:** Summary statistics of observations at baseline and for each expiratory resistance phase in 10 subjects.

	Baseline	Initial strain	Early strain	Late strain	Recovery
Bulk BTP amplitude, median (IQR) (µm)	11.5 (5.7, 14.3)	14.2 (7.0, 20.3)^*^	11.5 (6.8, 16.5)	8.9 (5.6, 15.2)	14.6 (5.8, 16.5)
MAP, mean (SD) (mmHg)	86.8 (10.8)	94.3 (9.4)	86.6 (9.6)	87.8 (8.8)	90.1 (11.7)
PP, mean (SD) (mmHg)	57.7 (9.9)	53.6 (13.4)	54.8 (10.8)	52.6 (17.0)	57.6 (15.0)
HR, mean (SD) (bpm)	63.5 (13.0)	64.6 (11.1)	66.7 (10.7)	73.9 (12.7)^*^	60.8 (11.1)

*Note*: *Significant changes from baseline, based on adjusted *P*‐values from Dunn's or Dunnett's *post hoc* test. No sex‐specific differences were identified in bulk BTP amplitude or any other measured variable.

**FIGURE 4:**
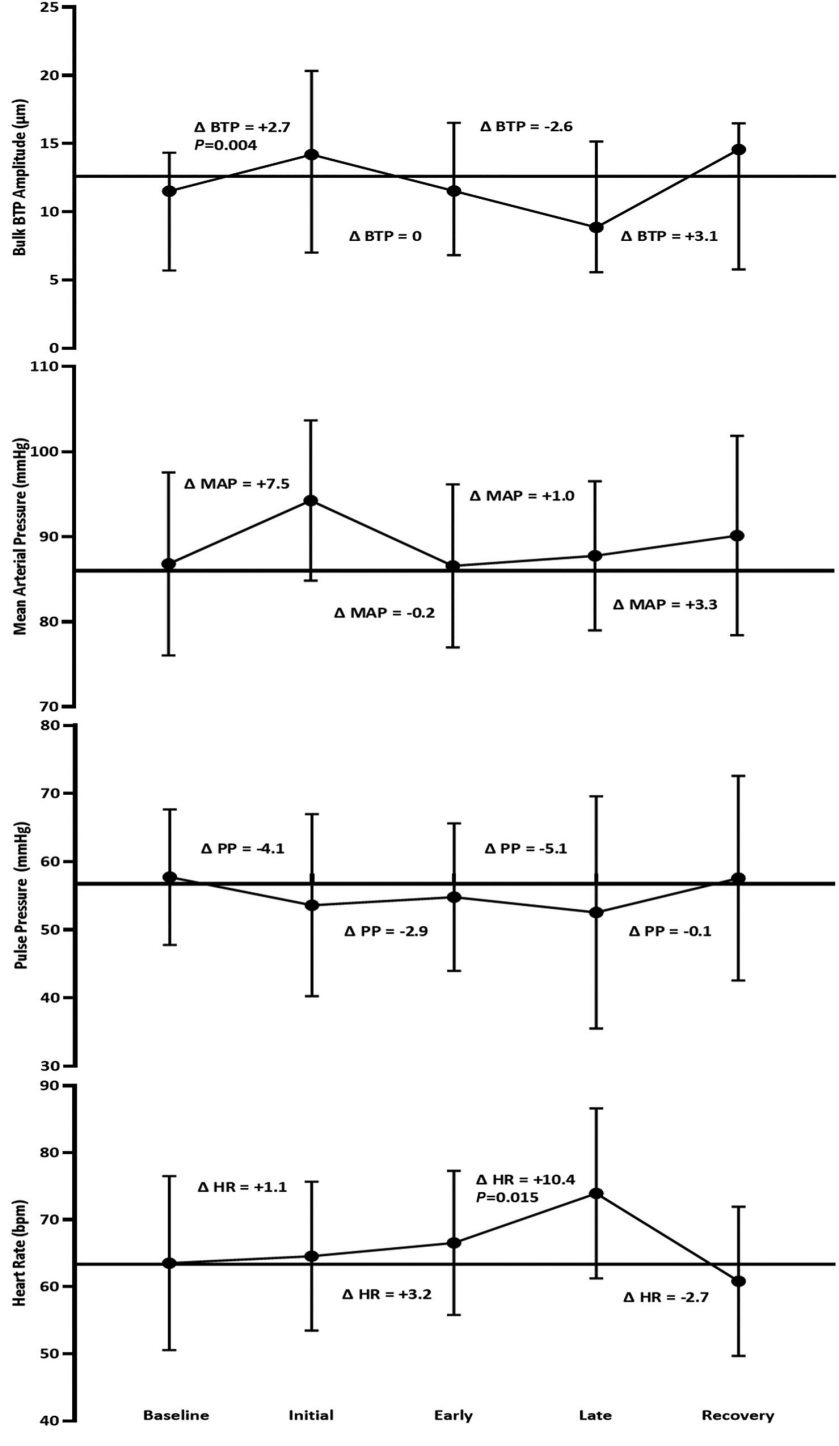
Summary of changes between baseline with each expiratory resistance phase and recovery in 10 healthy participants. Bulk brain tissue pulsation (BTP) amplitude is expressed as median and IQR values, with all other variables expressed as mean and SD. Estimated differences between baseline and each phase of the manoeuvre (including recovery) are labelled in the figure. Significant differences were based on adjusted *P*‐values from Dunn's or Dunnett's *post hoc* test.

For HR, the effect of time was also significant (*F*(4, 36) = 6.31, *P* = 0.009). HR increased significantly between baseline and the late phase by 10.4 bpm (95% CI: 2.18 to 18.61, *P* = 0.015). No significant differences were observed during the initial, early phase or recovery. No significant changes were observed in MAP or PP.

Individual changes between participants for all variables were also investigated. Bulk BTP amplitude, our brain pulsation parameter of interest, did not vary widely between subjects at baseline (∼2 to ∼18 µm). Figure 5 shows changes in each reported variable for all 10 participants at the initial, early and late phases of the manoeuvre and recovery, in comparison to baseline.

**FIGURE 5:**
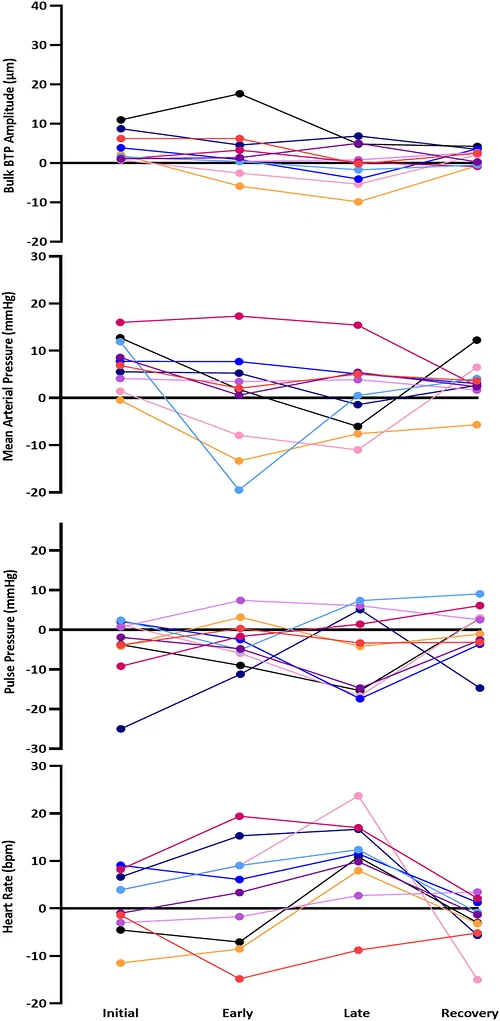
Summary of mean beat‐to‐beat changes between baseline and the initial, early and late phases of the manoeuvre and recovery in all measured variables for each of the 10 participants. Each participant is denoted by a different colour.

## 4 DISCUSSION

In this pilot healthy volunteer study, we characterised BTP responses during a low‐level expiratory resistance manoeuvre using TCTD. Systemic effects were confirmed by concurrent measurement of MAP, PP, and HR. To our knowledge, this is the first study to examine these responses using a real‐time ultrasound‐based measure of brain tissue motion. The principal finding was a small, yet significant, increase in bulk BTP amplitude during the first 5 s of a low‐level expiratory resistance manoeuvre. Although bulk BTP amplitude subsequently returned towards baseline and decreased below baseline during the late phase of the manoeuvre, these later changes were not statistically significant. A significant increase in HR was observed during the late phase of the manoeuvre, whilst no significant changes were identified in MAP or PP. The present study provides an exploratory characterisation of BTP responses during low‐level expiratory resistance, whilst the physiological mechanisms underlying these responses remain uncertain.

Several physiological factors may have contributed to the observed BTP responses, including changes in intracranial blood volume, vascular dynamics, venous return, intracranial compliance, cerebrospinal fluid dynamics and transmission of intrathoracic pressure to the arterial circulation, or a combination of these factors. However, these variables, in addition to ICP, were not directly measured, and therefore their relative contributions cannot be determined from the present data.

The potential collective influence of these factors on brain tissue motion is consistent with previous studies reporting alterations in brain displacement during interventions associated with changes in intracranial physiology (Holdsworth et al., 2016; Kumar et al., 2025; Saindane et al., 2018). Reduced brain tissue displacement measured using amplified magnetic resonance imaging has been observed in the pons, cerebellum, and spinal cord during a 25‐s Valsalva manoeuvre compared to free breathing (Holdsworth et al., 2016), although this study included only three participants. Conversely, increased brain displacement has been observed following lumbar puncture, an intervention associated with reduced ICP (Kumar et al., 2025; Saindane et al., 2018). In patients with idiopathic intracranial hypertension, mean pontine displacement increased to values similar to those of healthy subjects following cerebrospinal fluid removal (Saindane et al., 2018). These observations suggest that brain motion is influenced by a complex interplay of intracranial physiological processes.

Systemic effects served as supportive indicators relevant for interpreting changes in bulk BTP amplitude. An initial increase in MAP was observed alongside a significant increase in bulk BTP amplitude. However, given that previous work has demonstrated that MAP has no discernible impact on bulk BTP amplitude in isolation (Nicholls et al., 2023), it is unlikely that MAP was the primary driver of the observed changes in BTPs.

A reduction in PP at the late phase of the manoeuvre was observed alongside a reduction in bulk BTP amplitude. Given that PP is a recognised determinant of bulk BTP amplitude (Nicholls et al., 2023), this may partly explain the observed decrease in the late phase, although it is unlikely to explain all the observed changes in bulk BTP amplitude during the manoeuvre, suggesting that additional intracranial factors may contribute. This reduction in PP also occurred alongside a significant increase in HR. However, the specific influence of HR on bulk BTP amplitude remains unclear, as previous studies have not isolated HR from concurrent haemodynamic changes.

Although the magnitude of changes was small, our findings suggest that TCTD is capable of detecting physiological changes occurring during low‐level expiratory resistance. Notably, no previous studies have used TCTD to investigate BTP responses during a low‐level expiratory resistance manoeuvre. TCTD offers several practical advantages, providing real‐time information on intracranial haemodynamics, whilst being non‐invasive, portable and not requiring specialist operator expertise, supporting its potential suitability as a clinical tool for assessing intracranial dynamics.

Alterations in BTPs have previously been reported in a range of pathological conditions associated with disturbed ICP, including stroke, Chiari malformation 1 and idiopathic syringomyelia (Abderezaei et al., 2024; Ince et al., 2020, 2022; Li et al., 2015). However, the relationship between ICP and brain tissue motion remains poorly understood, and further work is needed to determine whether BTP measurements have a role in ICP monitoring. Improved understanding of this relationship may provide insight into the mechanisms that act to protect the brain under conditions of raised ICP, and how these mechanisms may be impaired in disease. Measurement of brain displacement in patients undergoing long‐term ICP monitoring would help to further elucidate this relationship.

As with most physiological measurement studies, our study design was associated with several limitations. The findings of this study should be interpreted as exploratory and hypothesis‐generating. Firstly, as this healthy volunteer study was a pilot study, our sample size was small (10 participants). This was because the study was designed as a proof‐of‐concept study to investigate the effect of a low‐level expiratory manoeuvre on bulk BTP amplitude, with the aim of identifying trends and understanding signal detectability, rather than formulating statistically powered conclusions. The median age was also skewed toward younger participants, for which age is a known predictor of bulk BTP amplitude at the forehead (Turner et al., 2020). Future work should use a larger sample size with older participants and investigate whether vascular and parenchymal stiffness are associated with differences in bulk BTP amplitude.

Reported changes in bulk BTP amplitude across the cohort during the manoeuvre were also small. Despite this, the observed changes were measurable (between ∼0.2 and ∼17.6 µm). However, it is worth noting that some of the observed changes were close to Brain TV's accuracy threshold of 1.6 µm (Nicholls et al., 2025), and should be interpreted with caution. Applying longitudinal modelling approaches to characterise within‐participant trajectories in each physiological variable will also be required to confirm the observed changes in bulk BTP amplitude.

We used a whistle containing a low‐level PEEP valve that was mechanically limited to a maximum pressure of ∼14.7 mmHg, which is substantially lower than the pressures commonly employed during standardised Valsalva manoeuvres, which are often performed at ∼40 mmHg. Consequently, the physiological perturbation induced by the present protocol may have been modest. In addition, the whistle did not undergo external validation for pressure consistency, and variation in manoeuvre execution between participants could not be directly controlled, potentially leading to variability in the magnitude of the physiological stimulus. Therefore, the magnitude and consistency of the manoeuvre may have varied across participants due to the differences in manoeuvre execution, which could not be directly controlled within the study. This may explain the relatively small magnitude of the observed BTP changes and may have reduced our ability to detect consistent responses across all phases of the manoeuvre. Furthermore, as ICP was not directly measured, the extent to which the manoeuvre altered ICP or other intracranial physiological variables cannot be determined. Therefore, the present findings should be interpreted as describing BTP responses during low‐level expiratory resistance rather than establishing a causal relationship between BTPs and ICP. Future studies should investigate BTP responses across a wider range of physiological stimuli and incorporate direct ICP measurements alongside complementary physiological monitoring to characterise the relationship between BTPs and intracranial dynamics.

Despite a significant increase in HR observed during the late phase of the manoeuvre, its independent effect on bulk BTP amplitude cannot be determined from this dataset. As HR was not analysed in isolation from concurrent haemodynamic changes, it remains unclear whether HR contributes to the observed BTP dynamics. Therefore, the increase in HR represents a potential confounding factor that should be considered when interpreting the relationship between expiratory resistance and BTP dynamics. Changes in venous outflow on BTPs have also not been characterised, suggesting additional imaging (magnetic resonance imaging) in combination with TCTD is needed to visualise changes in cerebral blood flow.

We analysed responses in bulk BTP amplitude and systemic parameters using fixed 5‐s time windows, which may not capture the dynamic physiological responses occurring during expiratory resistance. Future studies incorporating a thoracic impedance probe could help more accurately define the timing of physiological changes.

The release phase of the expiratory resistance was not analysed due to its short interval and increased susceptibility to motion artefacts associated with active movement of each participant. The likelihood of increased motion artefacts increasing noise in our data at the end of the strain was also why recovery was analysed 5 s after the strain was relieved. Whilst these adjustments were necessary to minimise motion‐related noise, they could be identified as limitations of our study.

Previous work has highlighted that brief hypocapnia induces an increase in BTPs (Alharbi et al., 2020). However, end‐tidal carbon dioxide was not recorded in this study as the placement of the nasal cannula would likely block participants from manoeuvring the whistle towards their mouth at the correct time, subsequently increasing noise in the BTP signal. Therefore, we acknowledge that without directly measuring end‐tidal carbon dioxide, the contribution of carbon dioxide on the observed reduction in bulk BTP amplitude cannot be excluded.

We acknowledge that cerebral blood velocity was not measured in this study. Due to the low pulse repetition frequency of the Brain TV device, simultaneous measurement of BTPs using Brain TV and cerebral blood velocity using transcranial Doppler is not yet possible. Future studies should aim to utilise measurement approaches that enable concurrent assessment of both signals to further our understanding of the relationship between BTPs and cerebral blood velocity.

The present analysis utilised a bulk BTP signal averaged across depths, which may obscure potential regional differences in tissue pulsatility. It is plausible that deeper and more superficial brain tissue regions exhibit differing sensitivity to ICP. Future studies should incorporate depth‐resolved analysis to provide further insight into spatial variation in the BTP signal.

In conclusion, this pilot healthy volunteer study demonstrated a dynamic bulk BTP response during a low‐level expiratory resistance manoeuvre, characterised by a significant initial increase and followed by a non‐significant decrease as the manoeuvre continued. These findings suggest that measurable changes in bulk BTP amplitude occur during low‐level expiratory resistance. The physiological mechanisms underlying these observations remain uncertain, as ICP and other intracranial physiological variables were not directly measured. Future studies incorporating direct ICP measurements, including patients undergoing long‐term ICP monitoring, and other complementary physiological monitoring, are needed to characterise the relationship between BTPs and intracranial dynamics.

## AUTHOR CONTRIBUTIONS

This study was performed at the Bubble Test Clinic, University of Leicester Medicine Department at Leicester Royal Infirmary. Jennifer K. Nicholls: Conception or design of the work, acquisition or analysis or interpretation of data for the work, drafting the work or revising it critically for important intellectual content. Emma M. L. Chung: Conception or design of the work, acquisition or analysis or interpretation of data for the work, drafting the work or revising it critically for important intellectual content. Jonathan Ince: Acquisition or analysis or interpretation of data for the work, drafting the work or revising it critically for important intellectual content. Edward Pallett: Drafting the work or revising it critically for important intellectual content. Jatinder S. Minhas: Acquisition or analysis or interpretation of data for the work, drafting the work or revising it critically for important intellectual content. Andrea Lecchini‐Visintini: Acquisition or analysis or interpretation of data for the work, drafting the work or revising it critically for important intellectual content. All authors have read and approved the final version of this manuscript and agree to be accountable for all aspects of the work in ensuring that questions related to the accuracy or integrity of any part of the work are appropriately investigated and resolved. All persons designated as authors qualify for authorship, and all those who qualify for authorship are listed.

## CONFLICT OF INTEREST

None declared.

## GENERATIVE AI STATEMENT

No generative AI tools were used in the preparation of this manuscript.

## Data Availability

Data are available upon reasonable request to the corresponding author.
